# Diabetic cardiomyopathy in Zucker diabetic fatty rats: the forgotten right ventricle

**DOI:** 10.1186/1475-2840-9-25

**Published:** 2010-06-15

**Authors:** Charissa E van den Brom, Joanna WAM Bosmans, Ronald Vlasblom, Louis M Handoko, Marc C Huisman, Mark Lubberink, Carla FM Molthoff, Adriaan A Lammertsma, Margriet D Ouwens, Michaela Diamant, Christa Boer

**Affiliations:** 1 Department of Internal Medicine/Diabetes Centre, VU University Medical Centre, Amsterdam, the Netherlands; 2 Laboratory for Physiology, VU University Medical Centre, Amsterdam, the Netherlands; 3 Department of Pulmonology, VU University Medical Centre, Amsterdam, the Netherlands; 4 Department of Nuclear Medicine & PET Research, VU University Medical Centre, Amsterdam, the Netherlands; 5 Department of Anaesthesiology, VU University Medical Centre, Amsterdam, the Netherlands; 6 German Diabetes Centre, Institute for Clinical Biochemistry and Pathobiochemistry, Düsseldorf, Germany

## Abstract

**Background:**

In patients with myocardial infarction or heart failure, right ventricular (RV) dysfunction is associated with death, shock and arrhythmias. In patients with type 2 diabetes mellitus, structural and functional alterations of the left ventricle (LV) are highly prevalent, however, little is known about the impact of diabetes on RV characteristics. The purpose of the present study was to investigate whether LV changes are paralleled by RV alterations in a rat model of diabetes.

**Methods:**

Zucker diabetic fatty (ZDF) and control (ZL) rats underwent echocardiography and positron emission tomography (PET) scanning using [^18^F]-2-fluoro-2-deoxy-D-glucose under hyperinsulinaemic euglycaemic clamp conditions. Glucose, insulin, triglycerides and fatty acids were assessed from trunk blood. Another group of rats received an insulin or saline injection to study RV insulin signaling.

**Results:**

ZDF rats developed hyperglycaemia, hyperinsulinaemia and dyslipidaemia (all p < 0.05). Echocardiography revealed depressed LV fractional shortening and tricuspid annular plane systolic excursion (TAPSE) in ZDF vs. ZL rats (both p < 0.05). A decrease in LV and RV insulin-mediated glucose utilisation was found in ZDF vs. ZL rats (both p < 0.05). LV associated with RV with respect to systolic function (r = 0.86, p < 0.05) and glucose utilisation (r = 0.74, p < 0.05). TAPSE associated with RV MRglu (r = 0.92, p < 0.05) and *M*-value (r = 0.91, p < 0.0001) and RV MRglu associated with *M*-value (r = 0.77, p < 0.05). Finally, reduced RV insulin-stimulated phosphorylation of Akt was found in ZDF vs. ZL (p < 0.05).

**Conclusions:**

LV changes were paralleled by RV alterations in insulin-stimulated glucose utilisation and RV systolic function in a rat model of diabetes, which may be attributed to ventricular interdependence as well as to the uniform effect of diabetes. Since diabetic patients are prone to develop diabetic cardiomyopathy and myocardial ischaemia, it might be suggested that RV dysfunction plays a central role in cardiac abnormalities in this population.

## Background

Although been underestimated in the past, the contribution of right ventricular (RV) function to overall myocardial contractility is considerable. RV dysfunction is relevant in a variety of disease states, like pulmonary hypertension, coronary heart disease and heart failure. RV dysfunction is an independent predictor of outcome in patients with myocardial infarction [1] and heart failure [2,3]. Patients with type 2 diabetes are at increased risk of myocardial infarction [4], however, not much is known on RV involvement in these patients.

While left ventricular (LV) impairment is a common complication of type 2 diabetes mellitus (T2DM), the number of studies focusing on the impact of T2DM on RV function is limited and conflicting [5-8]. We [9] and others [10,11] have shown that alterations in LV energy metabolism, resulting from changes in substrate supply and utilisation, contribute to the development of myocardial dysfunction, but this association has not been established for the RV. Despite the association of RV dysfunction with aggravation of myocardial function and outcome in distinct cardiac diseases, its role in diabetic cardiomyopathy is unknown. The purpose of the present study was therefore to investigate whether LV changes are paralleled by RV alterations with respect to glucose utilisation and function in an experimental rat model of diabetes.

## Material and methods

### Animals

All experiments were approved by the Animal Care and Use Committee of the VU University, and were conducted following the European Convention for the Protection of Vertebrate Animals used for Experimental and Other Scientific Purposes, and the Dutch Animal Experimentation Act.

Male Zucker diabetic fatty (ZDF) rats (fa/fa; n = 12, Charles River Laboratories Brussels, Belgium) and age-matched Zucker lean control rats (ZL; +/+; n = 10) underwent echocardiography and positron emission tomography (PET) at 14 weeks of age. Trunk blood was collected for determinations of blood glucose, plasma insulin, fatty acid and triglyceride levels [12]. Hearts were removed and either snap-frozen in dry-ice-chilled isopentane or fixed in 4% formalin for further biochemical and histological analysis.

### Echocardiography

Echocardiographic analyses were performed as previously described [9,12]. Measured parameters for RV function were Doppler-derived cardiac output and tricuspid annular plane systolic excursion (TAPSE). Parameters for RV remodeling and pulmonary vascular remodeling were measured as RV end-diastolic diameter and pulmonary artery acceleration time normalised for cycle length (PAAT/cl), respectively [13]. Further, estimated RV systolic pressure (eRVSP) was determined by 142*e^(-11*[PAAT/cl]) ^[12].

### Positron emission tomography

LV and RV glucose metabolism were measured using [^18^F]-2-fluoro-2-deoxy-D-glucose (^18^FDG) PET under hyperinsulinaemic euglycaemic clamp conditions, a gold-standard estimate of whole-body insulin sensitivity [9]. Quantification was achieved with compartment analysis, based on a two-tissue compartment model [9].

### Histology

Sections of LV and RV were stained with hematoxylin-eosin for determination of cardiomyocyte cross-sectional area [12]. Picrosirius red staining was used for analysis of myocardial fibrosis. LV and RV fibrosis were expressed as the percentage tissue area positive for collagen, measured over minimally three randomly chosen areas per ventricle [13].

### Western blotting

A separate group of rats (n = 4, ZDF rats; n = 4, ZL rats) received insulin stimulation (10 U·kg^-1 ^BW insulin) or saline as control (n = 4, ZDF rats; n = 4, ZL rats) to study RV insulin signaling [9,14] using the following primary antibodies: phospho-Akt-Ser473, phospho-GSK3β-Ser9, GSK3β (all Cell signaling Technology, Beverly, MA) and Akt2 (Upstate, Lake Placid, NY).

RV substrate metabolism was studied using glucose transporter 4 (GLUT4; Abcam, Cambridge, MA), phospho-AMP activated protein kinase (AMPK)-Thr172, AMPK (both Cell signaling Technology, Beverly, MA) and pyruvate dehydrogenase kinase-4 (PDK4; Santa Cruz Biotechnology, Santa Cruz, CA) [8]. All signals were normalised to actin (Sigma, Saint Louis, Missouri) or total protein expression.

### Statistical analysis

All data are presented as mean ± SEM. Group comparisons were performed using student t-test or two-way ANOVA with Bonferroni post-hoc analysis. Correlations were calculated by the Pearson's test. p < 0.05 was considered as statistically significant.

## Results

Consistent with a diabetic phenotype, ZDF rats were obese, hyperglycaemic, hyperinsulinaemic and dyslipidaemic compared with ZL rats as shown in Table 1. The clamp-measured *M*-value was significantly lower in ZDF rats than in control rats (Table 1).

**Table 1:** Characteristics of ZL (+/+) and ZDF (*fa/fa*) rats at 14 weeks of age

	ZL	ZDF
Body weight at killing [g]	335 ± 4	355 ± 8*
Non-fasting blood glucose [mmol·L^-1^]	5.0 ± 0.1	20.9 ± 0.8*
Fasting insulin [pmol·L^-1^]	116.9 ± 11.9	330.7 ± 69.6*
Fasting triglycerides [mmol·L^-1^]	0.36 ± 0.02	1.41 ± 0.20*
Fasting fatty acids [mmol·L^-1^]	0.16 ± 0.03	0.38 ± 0.08*
*M*-value [mg·kg·min^-1^]	22.4 ± 1.0	8.9 ± 0.8*

Data are expressed as mean ± SEM, n = 8, * p < 0.05 vs. ZL

ZL, Zucker lean; ZDF, Zucker diabetic fatty

**Figure 1:**
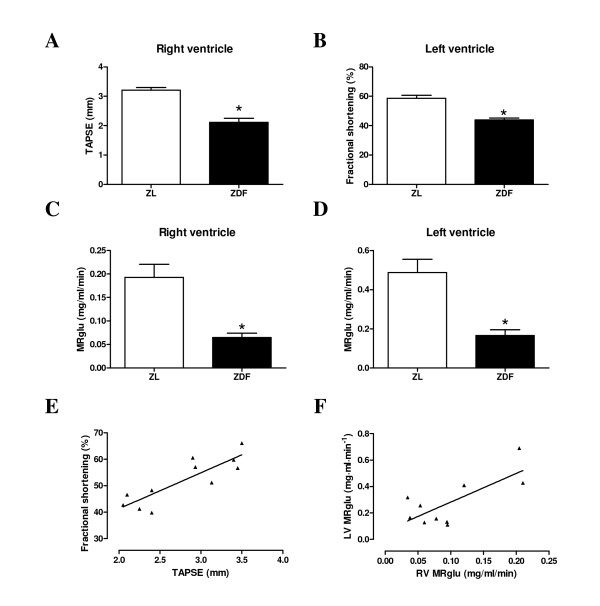
***In vivo *alterations in right and left ventricular glucose metabolism and function**. Relative change in systolic function of RV (tricuspid annulus systolic plane excursion; TAPSE) (**A**) and LV (fractional shortening) (**B**) and metabolic rate of glucose utilisation (MRglu) measured under hyperinsulinaemic euglycemic clamp conditions for RV (**C**) and LV (**D**) of ZL rats (white bars) and ZDF rats (black bars). Data are expressed as mean ± SEM, n = 4-9, * p < 0.05 vs. ZL rats. Relationship of RV systolic function (TAPSE) with LV systolic function (fractional shortening) (**E**; r = 0.86, p < 0.05, n = 11) and RV MRglu with LV MRglu (**F**; r = 0.74, p <0.05, n = 10).

**Figure 2:**
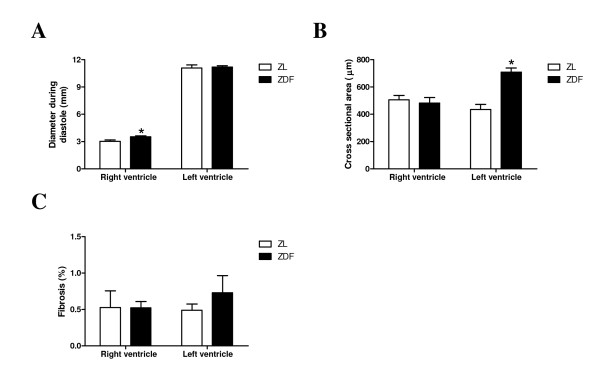
**Dilatation of the right ventricle and hypertrophy in the left ventricle**. Diastolic diameter of RV and LV (**A**), cross sectional area of individual cardiomyocytes in RV and LV (**B**) and quantification of fibrosis (**C**) of ZL rats (white bars) and ZDF rats (black bars). Data are expressed as mean ± SEM, n = 4-9, * p < 0.05 vs. ZL rats.

Echocardiography in ZDF rats showed a significant decreased in RV and LV systolic function compared to controls (Figure 1A and 1B), as measured by TAPSE and fractional shortening, respectively. Cardiac output remained unchanged in ZDF rats versus controls (96 ± 8 ml/min vs. 93 ± 6 ml/min for ZDF and ZL rats, respectively, n.s.). TAPSE associated positively with LV fractional shortening (r = 0.86, p < 0.05, Figure 1E). Interestingly, RV diastolic diameter was significantly increased, whereas LV diastolic diameter remained unchanged (Figure 2A). Furthermore, PAAT/cl showed no difference between the two experimental groups (0.17 ± 0.01 for ZDF rats vs. 0.18 ± 0.01 for ZL rats, n.s.). Estimated RVSP remained also unchanged between both groups (21.2 ± 1.9 mmHg for ZDF rats vs. 23.6 ± 2.1 mmHg for ZL rats, n.s.).

RV and LV metabolic rates of glucose utilisation (MRglu) measured under hyperinsulinaemic euglycaemic conditions were decreased by 66% and 60%, respectively, in ZDF versus ZL rats (Figure 1C and 1D). RV MRglu was positively associated with LV MRglu (r = 0.74, p < 0.05, Figure 1F).

TAPSE associated significantly with RV MRglu (r = 0.92, p < 0.05) and *M*-value (r = 0.91, p < 0.0001) and RV MRglu associated significantly with *M*-value (r = 0.77, p < 0.05).

RV weight, RV/(LV + septum) ratio, LV weight and lung weight were similar in ZDF and ZL rats (data not shown). Cross-sectional areas of individual RV cardiomyocytes were similar between both groups, whereas cross sectionals areas of individual LV cardiomyocytes were significantly increased (Figure 2B) in ZDF rats, suggesting different remodeling between ventricles. Further, RV and LV fibrosis remained unchanged between both groups (Figure 2C).

**Figure 3:**
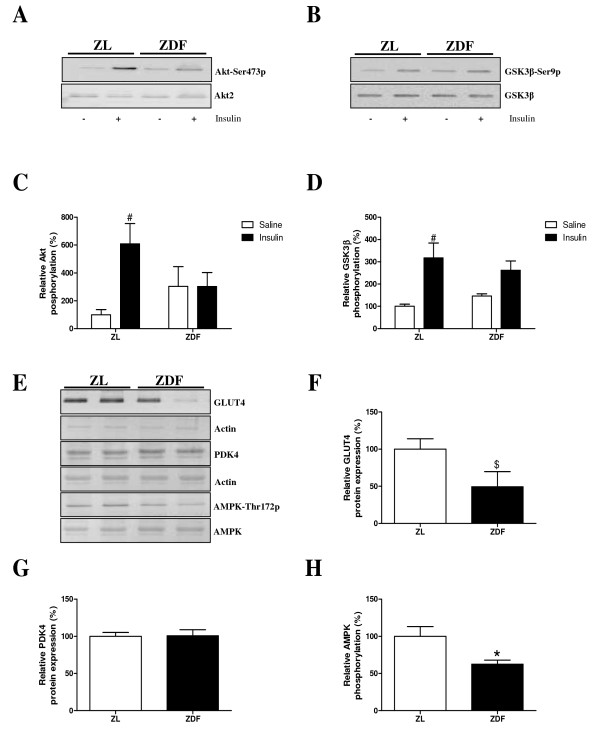
**Molecular changes in right ventricular insulin signaling**. Immunoblots showing Akt (**A**) and glycogen synthase kinase (GSK)3β (**B**) and glucose transporter (GLUT)4, pyruvate dehydrogenase kinase (PDK)4 and AMP activated kinase (AMPK) (**E**). Quantification of immunoblots showing relative phosphorylation of Akt (**C**) and GSK3β (**D**) after saline (-; open bars) or insulin (+; filled bars) injection in RV. Quantification of immunoblots showing relative protein expression of GLUT4 (**F**), PDK4 (**G**) and AMPK phosphorylation (**H**) in ZL rats (white bars) and ZDF rats (black bars). Data are expressed as mean ± SEM, n = 4, * p < 0.05 vs. ZL rats, $ p = 0.09 vs. ZL rats, # p < 0.05 insulin vs. saline effect.

Phosphorylation of Akt and GSK3β were studied to determine RV insulin sensitivity. ZDF and ZL hearts showed similar basal Akt and GSKβ phosphorylation. RV insulin-dependent phosphorylation of Akt-Ser473 was decreased in ZDF rats (two-way ANOVA interaction p = 0.05, Figure 3A and 3C). Further, insulin injection increased GSK3β phosphorylation significantly in ZL rats, but this increase was blunted in ZDF rats (Figure 3B and 3D).

Glucose transporter 4 (GLUT4) and AMP-activated kinase (AMPK) protein expression was decreased in ZDF rats compared to ZL rats, however, GLUT4 failed to reach significance (p = 0.09, Figure 3E, F and 3H). Further, pyruvate dehydrogenase kinase 4 (PDK4) protein expression remained unchanged between the groups (Figure 3G).

## Discussion

Using echocardiography and ^18^FDG PET, the main finding of this study was that diabetic cardiomyopathy in an experimental model of diabetes is characterised by RV and LV alterations. In particular, changes in RV function were associated with systemic insulin sensitivity and reduced RV insulin-stimulated glucose utilisation. The alterations in RV and LV insulin-stimulated glucose metabolism and systolic function may be attributed to ventricular interdependence, but also indicate that diabetes similarly affects both ventricles.

Impaired RV systolic function as reported in the present study is in agreement with data from Mittal [5] and Movahed *et al*. [8] who reported comparable results in T2DM patients. Others showed deterioration of RV diastolic function in these patients, whereas the association with RV systolic dysfunction was undecided ambivalent [6,7,15]. In addition, the metabolic alterations in the RV as found in the present study are consistent with previously reported findings in ^18^FDG PET studies of the LV in ZDF rats [9,16].

A restriction to glucose utilisation in the diabetic heart is the slow rate of glucose transport [17]. In line with previous reports of the LV [9,18,19], decreased GLUT4 expression was found, which is compatible with the *in vivo *change in RV insulin-stimulated glucose metabolism. Impaired insulin signaling is associated with alterations in glucose metabolism. Impaired systemic and RV insulin sensitivity was demonstrated by hyperinsulinaemic euglycaemic clamp and an impaired ability of insulin to phosphorylate Akt and GSK3β. Overall these molecular analyses are in line with the molecular changes found in the LV [9], suggesting the uniform effect of diabetes on the heart. Finally, an association was found between RV and LV changes with respect to systolic function and glucose metabolism.

Remodeling of the ventricles was found to be different despite comparable changes in function and metabolism between the ventricles. Despite the absence of fibrosis in both ventricles, LV remodeling in ZDF rats is indicated by hypertrophy of individual cardiomyocytes in the absence of LV dilatation. In contrast, RV remodeling in ZDF rats is indicated by dilatation of the RV in the absence of hypertrophy. This is in agreement with Kosmala *et al*. [7] who reported the absence of changes in RV free wall thickness.

Briefly, the following mechanisms can be hypothesized: 1) increased LV filling pressure is translated to the pulmonary circulation, 2) ventricular interdependence and 3) both ventricles are exposed to the metabolic abnormalities characterising T2DM. Measurements on afterload and RV remodeling remained unchanged; we can therefore conclude that there are no differences in pulmonary vascular resistance. Based on the geometry of the heart it is expected that there is ventricular interdependence. However, in a model of diet-induced obesity, impaired LV function in the absence of RV dysfunction was found (data not shown), which suggests that the effect of diabetes on RV function may be of greater importance than previously assumed. In combination with the *in vivo *and molecular changes found in RV glucose metabolism and insulin signaling, this suggests that diabetes affects both ventricles.

Collectively, diabetes seems to affect RV glucose metabolism and RV systolic function in similar proportions as in the LV, which could be due to ventricular interdependence as well as the uniform effect of diabetes on the heart.

## Conclusion

Whereas consequences of diabetes on the heart were previously restricted to the LV, this study showed that LV insulin-stimulated glucose utilisation and LV systolic function are paralleled by significant alterations in RV insulin-stimulated glucose utilisation and RV function in an experimental rat model of diabetes. RV dysfunction is associated with worse outcome in a variety of cardiovascular diseases, including myocardial infarction and heart failure. The negative association of RV function with prognosis has only been scarcely investigated in the diabetic population. However, since diabetic patients are prone to develop diabetic cardiomyopathy and myocardial ischaemia [4], it might be suggested that RV dysfunction plays a central role in cardiac abnormalities in this population. This warrants further research with respect to the role of RV function in diabetic cardiomyopathy and its relation to patient outcome in diabetes.

## Competing interests

The authors declare that they have no competing interests.

## Authors' contributions

CEvdB participated in performing the study, data analysis, statistics and writing the manuscript. JWAM and RV in part performed the study. MLH contributed in interpretation of data. MCH and ML in part performed data analysis. CFMM in part performed the study and contributed to the design of the study. AAL in part participated in the design of the study and reviewed/edited the manuscript. DMO participated in the design of the study and reviewed/edited the manuscript. MD supervised the study, participated in the design of the study and reviewed/edited the manuscript. CB supervised the study and wrote/reviewed/edited the manuscript. All authors read and approved the final manuscript.
